# Inhibitory Effect of Lotus Leaf-Enriched Flavonoid Extract on the Growth of HT-29 Colon Cancer Cells through the Expression of PI3K-Related Molecules

**DOI:** 10.1155/2022/6770135

**Published:** 2022-05-09

**Authors:** Chong Li, Zhanming Zhou, Xingyao Long, Yanni Pan, Rui Wang, Xiufeng Chen, Xin Zhao

**Affiliations:** ^1^Chongqing Collaborative Innovation Center for Functional Food, Chongqing University of Education, Chongqing 400067, China; ^2^Chongqing Engineering Research Center of Functional Food, Chongqing University of Education, Chongqing 400067, China; ^3^Chongqing Engineering Laboratory for Research and Development of Functional Food, Chongqing University of Education, Chongqing 400067, China; ^4^Department of Endocrine and Breast Surgery, The first Affiliated Hospital of Chongqing Medical University, Chongqing 400016, China; ^5^Gastrointestinal Cancer Center, Chongqing University Cancer Hospital, Chongqing 400030, China

## Abstract

**Objectives:**

Lotus leaf is rich in flavonoids, and this study is aimed at examining the inhibitory effect of lotus leaf-enriched flavonoid extract (LLEFE) on HT-29 colon cancer cells through phosphatidylinositol 3-kinase/protein kinase B (PI3K/Akt) expression regulation.

**Methods:**

Lotus leaves were extracted by ethanol and purified using FL-3 macroporous resin to create the LLEFE. HT-29 colon cancer cells were tested using various methods: their proliferation was observed by 3-(4,5)-dimethylthiahiazo(-z-y1)-3,5-di-phenytetrazoliumromide (MTT) assay, their survival status was observed by fluorescence staining, their oxidative stress level was observed by biochemical kits, and their mRNA expression was determined by quantitative polymerase chain reaction (qPCR) assay. Additionally, the composition of the flavonoids in lotus leaf was determined by HPLC.

**Results:**

The results showed that the proliferation of NCM460 normal human colon cells was not affected by 0–500 *μ*g/mL LLEFE but the proliferation of HT-29 human colon cancer cells decreased. LLEFE increased the LDH level in an HT-29 colon cancer cell culture medium; also increased the superoxide dismutase (SOD), catalase (CAT) activities, and glutathione (GSH) level in HT-29 cells; and decreased the malondialdehyde (MDA) level. Further experimental results showed that LLEFE upregulated the expression of *SOD1*, *CAT*, and *GSH* mRNA and downregulated the expression of *PI3K*, *Akt*, and *mammalian target of rapamycin (mTOR)* in HT-29 cells. The high-performance liquid chromatography (HPLC) results showed that kaempferin, hyperoside, astragaloside, phloridzin, and quercetin were the main chemical constituents of lotus leaf.

**Conclusion:**

Lotus leaves contain functional flavonoids that inhibit the proliferation of HT-29 colon cancer cells and regulate the expression of PI3K/Akt through five important chemicals.

## 1. Introduction

Lotus (*Nelumbo* spp.) is a perennial herbaceous aquatic plant from the Nelumbonaceae family. Its leaves are often dried and then brewed as a tea [1]. In traditional Chinese medicine (TCM), lotus leaf is used to clear away heat and toxins, cool the blood, and stop bleeding [2]. In traditional Chinese medicine, lotus leaf is used for relieving heat, invigorating the spleen and stomach, regulating defecation, and others [3]. Lotus leaf tea assists in a variety of health functions [4]. Studies have shown that flavonoids and alkaloids, which are active substances, are found in the highest concentration in lotus leaves. Lotus leaf-enriched flavonoid extract (LLEFE) decreases blood lipids, relieves cardiovascular diseases, and possesses antiallergy, antiaging, antibacterial, and antioxidant properties [5–9]. Lotus leaf can be obtained from a wide range of plants, which makes it convenient to use as a TCM.

Colon cancer is a malignant gastrointestinal cancer. Chronic colitis and colon polyps may become carcinogenic [10]. Therefore, in addition to drug treatments to improve the quality of life and prolong the life of patients with colon cancer, prevention of symptoms, such as chronic colitis and colonic polyps, can reduce colon cancer. Studies have shown that plant flavonoids have preventive and interventional effects on colitis and colon polyps. Flavonoids can prevent and fight cancer mainly through three avenues: antifree radical, direct inhibition of cancer cell growth, and anticarcinogenic factors [11]. Carcinogenic contributors such as physicochemical factors lead to the enrichment of free radicals in the body, cause lipid peroxidation, destroy cellular DNA, and cause cancer. Flavonoids are free radical quenchers and antioxidants that can effectively prevent cell damage caused by lipid peroxidation and play an anticancer role [12].

In particular, flavonoids from natural plants can be added to functional foods that will then reduce the incidence of inflammation and cancer [11, 12]. Some studies have shown that a LLEFE can inhibit the proliferation of prostate cancer cells [13]. Animal research indicates that other plant flavonoids can directly inhibit liver and cervical cancer [14, 15]. *In vitro* experiments showed that different plant flavonoids exerted satisfactory specific inhibitory effects on different cancer cells. Tangerine peel inhibited the metastasis of mouse colon cancer, the growth of S180 cells, the growth and metastasis of Lewis lung cancer, and the growth of mouse melanoma B16. The combination of tangerine peel and hesperidin resulted in high inhibition of mouse WEHI3B (JCS) cells. It was also shown that hesperidin inhibits lung cancer [16]. The mechanism used by lotus leaf flavonoids may be similar and may act against cancer.

The expression of the PI3K/Akt signaling pathway is abnormally activated in some cancers. The two most widely discovered mechanisms of PI3K/Akt activation in human cancer are triggered by receptor tyrosine kinase (RTK) and somatic mutations in specific elements of the signaling pathway [17]. Stimulation and promotion of the PI3K pathway may negatively impact cancer treatment, and therefore, inhibition of PI3K may inhibit cancer development [18]. Abnormal stimulation of the PI3K/Akt pathway is associated with tumor growth and angiogenesis. The loss of PTEN function, which is usually seen in human tumors, leads to the stimulation of the PI3K/Akt pathway [19]. The key expression of the PI3K/Akt pathway results in clinically targeted therapeutic effects, including those that affect PI3K, Akt, and mTOR [20]. In this study, we verified that HT-29 colon cancer cells were inhibited by LLEFE via regulation of the PI3K/Akt signaling pathway *in vitro.* We also elucidated the relationship between the active components of LLEFE and the regulation of the PI3K/Akt signaling pathway.

## 2. Materials and Methods

### 2.1. LLEFE Extraction

To prepare the herb, 250 g of dried lotus leaves (Anhui Shoufeng Biotechnology Co. Ltd., Haozhou, Anhui, China) were weighed, crushed, and added to a 5 L beaker. Then, 4 L of 70% ethanol was added according to a 1 : 20 ratio of lotus leaves to ethanol (*w*/*v*). The beaker was placed in a water bath pot at 60°C for 3 h. The beaker was then removed and cooled. The mixture was pumped and filtered to obtain the lotus leaf-enriched flavonoid extract (LLEFE). The LLEFE crude extract was poured into a glass column containing FL-3 macroporous resin and filtered (Jiangsu Hecheng New Material Co. Ltd., Nanjing, Jiangsu, China). The filtrate from the first pass through the resin was discarded. After all of the extract was passed through the column, the glass column was eluted with 70% ethanol until the resin became colorless. The elution solution underwent rotary evaporation to remove the ethanol, and finally, the LLEFE was obtained [21].

### 2.2. Cancer Cell Culture

HT-29 human colon cancer cells and NCM460 normal human colon cells (Cyagen Biotechnology Co. Ltd., Taicang, Jiangsu, China) were used in this experiment. To prepare the culture medium, 10% fetal bovine serum (FBS) (*v*/*v*) and 1% penicillin-streptomycin (*v*/*v*) were added to RPMI-1640 medium (Solarbio Life Sciences, Beijing, China). HT-29 and NCM460 cells were grown in this medium at 37°C under 5% CO_2_. The medium was changed every two days. The experimental group was divided into control and LLEFE groups. The control group cells were not treated with LLEFE, but the LLEFE groups were treated with 0–500 *μ*g/mL LLEFE.

### 2.3. Cell Proliferation Detection by MTT Assay

For the MTT assay, 180 *μ*L of 1.0 × 10^4^/mL cells were seeded into 96-well plates. The cells were incubated at 37°C under a 5% CO_2_ atmosphere for 24 h. After the cells adhered to the plate walls, an additional 20 *μ*L of different concentrations of LLEFE solution was added to each well of the treatment group and the control group was treated with no LLEFE. After incubation for 48 h, the medium was discarded and 200 *μ*L of MTT reagent (the MTT reagent was dissolved in RPMI-1640 medium at a final concentration of 5 mg/mL; Solarbio Life Sciences, Beijing, China) was added to each well. After incubation for 4 h, the supernatant was discarded and 200 *μ*L of dimethyl sulfoxide was then added to each well. The OD value of each well was measured at 490 nm, and the inhibition rate of cell proliferation was calculated. Proliferation inhibition rate (%) = (control well OD − sample well OD)/control well OD × 100 [22].

### 2.4. Determination of Lactate Dehydrogenase (LDH), Superoxide Dismutase (SOD), Malondialdehyde (MDA), Glutathione (GSH), and Catalase (CAT) Levels in HT-29 Colon Cancer Cells

After the cells were treated according to the abovementioned methods, HT-29 human colon cancer cells were treated with LLEFE for 48 h. The medium was analyzed to determine the LDH levels as per the kit's instructions. Then, the cells were lysed by ultrasonication in an ice-water bath (vibrated for 3–5 s, repeated three times at 4 s intervals). The SOD, CAT activities, MDA, and GSH levels in the cells were also determined according to the instructions (Nanjing Jiancheng Bioengineering Institute, Nanjing, Jiangsu, China).

### 2.5. Observation of HT-29 Colon Cancer Cell Survival

To measure cell survival, 5 × 10^6^ cells were seeded in 6-well plates, cultured for 48 h, and then treated with LLEFE. HT-29 cell survival was observed under an inverted fluorescence microscope with a calcein-acetoxymethyl (AM)/propidium iodide (PI) double-staining kit. The living cells were labeled with green fluorescence, and the dead cells were labeled with red fluorescence (Olympus IX71 microscope, Tokyo, Japan).

### 2.6. qPCR Experiment

The total RNA was extracted using a kit (BaiMaiKe Technologies, Beijing, China), and the RNA concentration and purity were determined by a microspectrophotometer. Reverse transcription of RNA into cDNA was performed using a RevertAid First-Strand cDNA synthesis kit. Hieff™ qPCR SYBR® Green Master Mix (High Rox Plus) (Yeasen Technologies, Shanghai, China) and a real-time PCR system (StepOnePlus, Thermo Scientific, Bellefonte, PA, USA) were used to measure the target gene expression levels (Table 1). PCR was performed under the following conditions: denaturation was performed at 95°C for 3 min and then 60 cycles at 95°C for 15 s each, annealing was performed at 5°C for 30 s, denaturation was performed at 95°C for 30 s, and annealing was performed with 55 cycles for 35 s each. *β*-Actin was used for target gene expression, and the relative strength of expression was computed using the 2^−ΔΔ*Ct*^ method [22].

### 2.7. HPLC Assay

HPLC was used to determine the composition of the compounds in the LLEFE. The chromatographic conditions were as follows (UltiMate 3000 HPLC system, Thermo Fisher Scientific). A C18 chromatographic column (4.6 mm × 150 mm, 2.6 *μ*m, Thermo Fisher Scientific) was used with mobile phase A of 100% methanol and mobile phase B of 0.5% acetic acid. The flow rate was 0.5 mL/min, and the column temperature was 30°C. A UV-Vis detector was used with a detection wavelength of 285 nm, and the injection volume was 10 *μ*L. The amount of each LLEFE component was calculated using an external standard method: Mx = Cr × Ax/Ar × *C*, where *Mx* (mg/g) represents the component content, Cr (mg/mL) denotes the mass concentration of the standard, Ax denotes the peak area of the sample, Ar denotes the peak area of the standard, and *C* (1.0 mg/mL) denotes the concentration of the sample stock solution.

### 2.8. Statistical Analysis

SPSS 17.0 and GraphPad-Prism 7 software were used to analyze the data. Three parallel experiments were carried out throughout the study, and the experimental results were then averaged, and the standard deviation was calculated and expressed as the average ± standard deviation (SD). One-way ANOVA and *t*-test were used to analyze whether there was a statistical difference at the level of *P* < 0.05.

## 3. Results

### 3.1. Effect of LLEFE on Cell Proliferation

**Figure 1:**
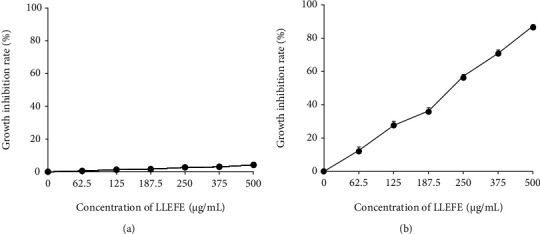
Effects of lotus leaf-enriched flavonoid extract (LLEFE) on (a) NCM460 normal human colon cells and (b) HT-29 human colon cancer cells proliferation.

Figure 1 shows that 0–500 *μ*g/mL of LLEFE exhibited only minor effects on the growth and proliferation of NCM460 normal human colon cells. At the same concentration, LLEFE inhibited the growth and proliferation of HT-29 human colon cancer cells and the inhibition rate increased with the increase in LLEFE concentration. At 125, 250, and 500 *μ*g/mL concentrations, LLEFE inhibited the proliferation of HT-29 human colon cancer cells by 19.98%, 55.85%, and 83.51%, respectively (Table 2). Based on the abovementioned experimental results, 125, 250, and 500 *μ*g/mL concentrations of LLEFE were used thereafter.

### 3.2. LDH Levels in HT-29 Colon Cancer Cell Culture Medium

**Figure 2:**
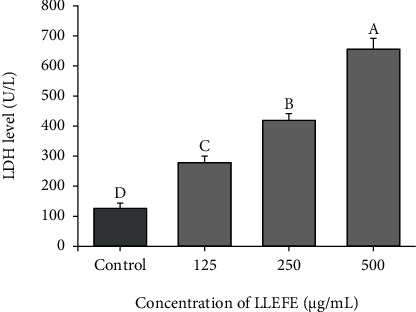
Effect of lotus leaf-enriched flavonoid extract on the LDH level of HT-29 colon cancer cell culture medium. Duncan multiple range test showed that ^a–d^ of different letters showed significant difference in the mean value of each group (*P* < 0.05).

As shown in Figure 2, the LDH level in the cell culture fluid of the control group was the lowest (125.62 U/L). As the concentration of LLEFE increased, the LDH level in the cell culture fluid also increased. The LDH levels in the culture medium of HT-29 colon cancer cells were 278.36, 418.76, and 655.87 U/L after treatment with LLEFE at 125, 250, and 500 *μ*g/mL, respectively.

### 3.3. SOD, MDA, GSH, and CAT Levels in HT-29 Colon Cancer Cells

As shown in Table 3, after 125, 250, and 500 *μ*g/mL of LLEFE treatment, the SOD and CAT enzymatic activities and GSH levels in all three treatment groups were significantly increased (*P* < 0.05), whereas the MDA level was significantly decreased (*P* < 0.05).

### 3.4. HT-29 Colon Cancer Cell Survival Status

**Figure 3:**
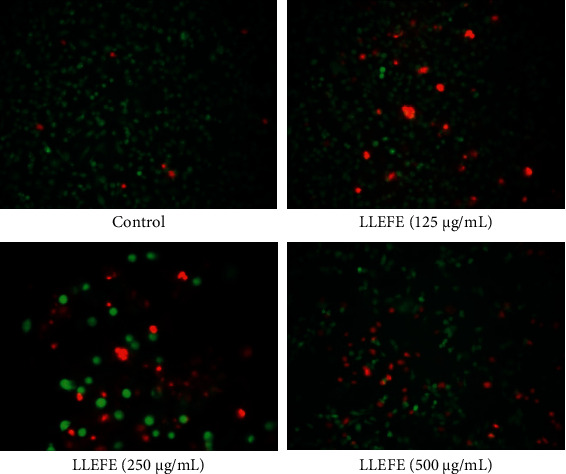
Effects of lotus leaf-enriched flavonoid extract on the survival of HT-29 colon cancer cells (200x); living cells were labeled with green fluorescence and dead cells with red fluorescence.

As shown in Figure 3, the HT-29 cell survival status was observed under an inverted fluorescence microscope. Control HT-29 cells were numerous, full, polygonal, and abundantly cytoplasmic, with numerous intense cells visible by fluorescent staining. HT-29 cells treated with LLEFE appeared shrunken and damaged, in decreased numbers, or dead. An increase in the concentration of LLEFE decreased the number of HT-29 cells and aggravated cell injury and death. These observations indicated that LLEFE inhibited the growth of HT-29 cancer cells.

### 3.5. mRNA Expression of HT-29 Colon Cancer Cells

**Figure 4:**
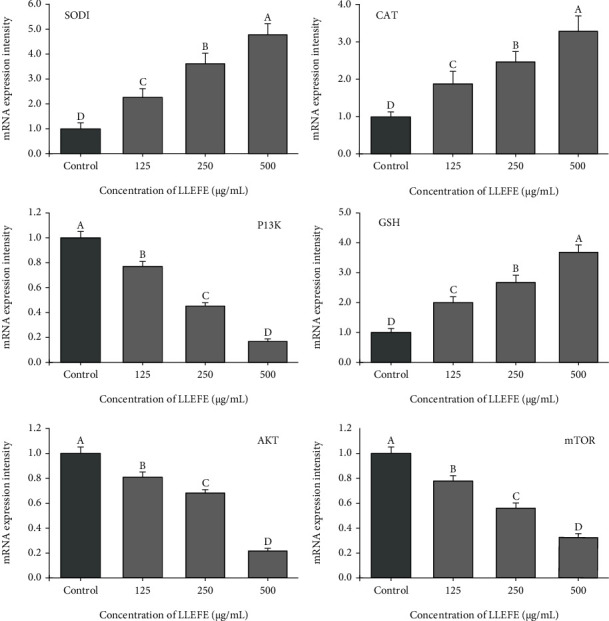
Effect of lotus leaf-enriched flavonoid extract on mRNA expression of HT-29 colon cancer cells. Duncan multiple range test showed that ^a–d^ of different letters showed significant difference in the mean value of each group (*P* < 0.05).

As shown in Figure 4, the control HT-29 cells showed the weakest *SOD1*, *CAT*, and *GSH* mRNA expression and the strongest *PI3K*, *Akt*, and *mTOR* mRNA expression. LLEFE was able to significantly (*P* < 0.05) upregulate SOD1, CAT, and GSH and significantly (*P* < 0.05) downregulate *PI3K*, *Akt*, and *mTOR* expression in HT-29 colon cancer cells. Moreover, with increasing LLEFE concentration (500 *μ*g/mL), there was stronger expression of *SOD1* (4.739-fold of the control), *CAT* (3.262-fold of the control), and *GSH* (3.687-fold of the control), while there was weaker expression of *PI3K* (0.176-fold of the control), *Akt* (0.218-fold of the control), and *mTOR* (0.333-fold of the control).

### 3.6. Analysis of LLEFE Composition

**Figure 5:**
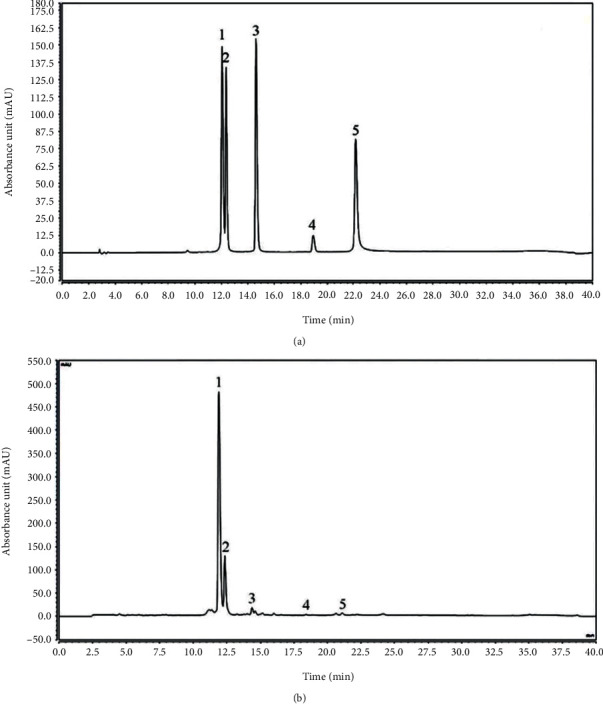
Analysis of flavonoids in lotus leaves. (a) Standard chromatograms; (b) lotus leaf-enriched flavonoid extract chromatograms. 1: kaempferin; 2: hyperoside; 3: astragaloside; 4: phloridzin; 5: quercetin.

As shown in Figure 5, the main chemical components in LLEFE were kaempferin, hyperoside, syringin, phlorizin, and quercetin. The amount of kaempferol was the highest at 77.62 mg/g, and the amounts of hyperoside, syringin, phlorizin, and quercetin were 35.12, 10.06, 0.31, and 0.12 mg/g, respectively.

## 4. Discussion

There was no effect or a reduced toxic effect of the active substance on normal somatic cells [23]. We were able to explore the mechanism of LLEFE's induction of cancer cell apoptosis. Under normal physiological conditions, the LDH content in body fluids is low and intracellular LDH is released only after cell membrane rupture [24]. Therefore, HT-29 cancer cells were damaged by LLEFE. A large amount of LDH was released into the medium after the membrane of HT-29 cells was broken and HT-29 cancer cells were more damaged with increasing LLEFE concentration.

Studies have shown that fairly high ROS levels exist in cancer cells, which often aid in cancer cell growth and proliferation [25]. As the main endogenous antioxidant in the body, SOD scavenges excessive ROS, decreases the mitochondrial damage in normal cells, and maintains body homeostasis [26]. GSH and CAT are also important antioxidants in the body and inhibit normal cellular damage caused by ROS [25]. MDA is a product of oxidative damage that can reveal the extent of cell damage [27]. It was thus suggested that the oxidative stress response in HT-29 cancer cells under LLEFE was inhibited, which inhibits the normal growth and proliferation of cancer cells.

Some studies have shown that the PI3K/Akt signaling pathway plays an important role in colorectal carcinogenesis by inducing tumor cell growth, differentiation, and vascularization, thus serving as a novel therapeutic target for colorectal cancer intervention [28]. Both PI3K and mTOR inhibitors suppress cancer cells under laboratory conditions, and some of these less toxic inhibitors have been validated in clinical trials and thus have the potential to act as promising new anticancer agents [29]. mTOR is a key kinase downstream of PI3K/Akt that plays a role in regulating cancer cell growth, proliferation, survival, and even metastasis [30]. PI3K, Akt, and mTOR expression can inhibit cancer cell proapoptotic factors in the body and activate anticancer cell apoptosis factors to promote cancer initiation and progression. PI3K-Akt-mTOR inhibits the activity of proapoptotic factors by phosphorylation while activating antiapoptotic factors. Studies have shown that the PI3K-Akt-mTOR signaling pathway plays a role in cancer cell proliferation. Deregulation of the PI3K-Akt-mTOR pathway may cause cancer recurrence and metastasis [31].

Additional studies have found that PI3K/Akt pathway activation promotes colorectal cancer cell proliferation and PI3KCA mutations are a common cause of this pathway activation. Clinical and experimental studies have shown that PI3KCA mutations may play an important role in the development of microsatellite instability (MSI) colorectal cancer and regulation of the PI3K/Akt pathway can effectively inhibit PI3KCA mutations [32]. A colorectal cancer study found that the PI3K/Akt signaling pathway induced angiogenic gene expression, promoted the formation of colon cancer blood vessels, and promoted tumor vascularization and metastasis [33]. More importantly, PI3K transmits mitotic signals to P70S6K1 through Akt and mTOR, which upregulates the translation of major cell cycle proteins, such as cyclin, and accelerates the cell cycle [34]. After PI3K activation promotes cell cycle progression, leading to the occurrence and development of colorectal cancer, inhibition of PI3K activity can lead to cell G1 arrest, inhibiting cell cycle progression and cancer cells [35].

Under normal physiological conditions, the body has the ability to withstand the stimulation of ROS. A low concentration of ROS *in vivo* is necessary for normal physiological metabolism. A large amount of ROS can stimulate an increase in antioxidant enzymes such as SOD and CAT that protect normal cells. When the body becomes cancerous, cancer cells are at a higher level of oxidative stress compared with normal cells and this oxidative stress causes normal cell damage. Oxidative stress also promotes the ROS level to stimulate and activate PI3K/Akt, thereby enhancing the proliferative ability of cancer cells [36]. In the present study, LLEFE effectively inhibited PI3K, Akt, and mTOR expression in the PI3K/Akt pathway and the process by which oxidative stress activates this pathway. This inhibited HT-29 cancer cell growth and proliferation.

Hydrogen peroxide (H_2_O_2_) is closely related to the level of LDH after cell injury, and the level intensity of H_2_O_2_ is highly consistent with the level of LDH. Studies have shown that lotus leaf flavonoids have a strong protective effect on normal cells. Under the same conditions, tea polyphenols and vitamin C have a strong inhibitory effect on the oxidative damage of normal cells, especially by adjusting the level of hydrogen peroxide. H_2_O_2_ can activate the expression of P13K/Akt [37], and it was also shown that wortmannin or LY294002, an inhibitor of P13K, promoted H_2_O_2_-regulated apoptosis. Therefore, the regulation of P13K/Akt expression can regulate the effect of H_2_O_2_ on cell injury [38].

LLEFE mainly contains five compounds (kaempferin, hyperoside, syringin, phlorizin, and quercetin) that are all flavonoids with promising antioxidant effects [39–43]. Hyperoside, phlorizin, and quercetin exert some confirmed anticancer effects [39–41]. Animal experiments showed that hyperoside can exert antioxidant effects through a PI3K/Akt regulation mechanism [42]. Another study confirmed that phlorizin can regulate the IRS2/PI3K/Akt pathway and reduce the accumulation of mitochondrial reactive oxygen species (ROS) to protect tissues [43]. Syringin can stimulate PI3K/Akt to regulate the body to maintain normalcy and avoid diseases including osteoporosis [44]. Quercetin can regulate PI3K/Akt-induced apoptosis of HL-60 cells and directly inhibit human promyelocytic leukemia cells [45]. The present study also confirmed that LLEFE containing these compounds inhibits the growth and proliferation of HT-29 colon cancer cells *in vitro.* LLEFE may effectively regulate the PI3K/Akt pathway, inhibit PI3K/Akt expression by the effective chemical compounds that it contains, control the level of oxidative stress, and inhibit colon cancer proliferation.

## 5. Conclusions

This study found that LLEFE inhibits HT-29 cancer cells without affecting normal cells, which may indicate anticancer specificity. At the same time, studies have shown that LLEFE regulates the expression and related expression of PI3K/Akt, which may be the key to its role. Further compositional analysis revealed that LLEFE contains five important flavonoid compounds and the combined effects of these compounds regulated the expression of the PI3K/Akt to inhibit HT-29 colon cancer cells. The effect and mechanism of LLEFE on colon cancer have only been preliminarily verified by *in vitro* experiments herein. Because it is a natural product and its action mechanism is complex, we only observed its effect on PI3K/Akt expression in this study and further study is required to elucidate its effect on additional pathways and its comprehensive effects. This study preliminarily verified the effects of LLEFE and provides a theoretical basis for further effective development of LLEFE as a new natural drug material.

## Figures and Tables

**Table 1:** Sequences of the primers used for this experiment.

Gene name	Sequence
*β-Actin*	F: 5′-TCAAGAAGGTGGTGAAGCAGG-3′
	R: 5′-AGCGTCAAAGGTGGAGGAGTG-3′
*CAT*	F: 5′-TGGAGCTGGTAACCCAGTAGG-3′
	R: 5′-CCTTTGCCTTGGAGTATTTGGTA-3′
*SOD1*	F: 5′-GGTGGGCCAAAGGATGAAGAG-3′
	R: 5′-CCACAAGCCAAACGACTTCC-3′
*GSH*	F: 5′-CAGTCGGTGTATGCCTTCTCG-3′
	R: 5′-GAGGGACGCCACATTCTCG-3′
*PI3K*	F: 5′-CTGCCTGCGACAGATGAGTG-3′
	R: 5′-TCCGATTACCAAGTGCTCTTTC-3′
*AKT*	F: 5′-AGCGACGTGGCTATTGTGAAG-3′
	R: 5′-GCCATCATTCTTGAGGAGGAAGT-3′
*mTOR*	F: 5′-ACAACTTTGGTATCGTGGAAGG-3′
	R: 5′-GCCATCACGCCACAGTTTC-3′

**Table 2:** Inhibitory effect of different concentrations of flavonoids from lotus leaf (LLEFE) on the proliferation of HT-29 human colon cancer cells.

Group	OD490 (concentration of LLPs, *μ*g/mL)			Cell growth inhibition rate (%)		
	125	250	500	125	250	500
Control	0.493 ± 0.006^a^			/		
LLEFE	0.357 ± 0.010^b^	0.211 ± 0.005^c^	0.063 ± 0.004^d^	27.59 ± 2.32^C^	57.10 ± 1.00^B^	87.20 ± 0.81^A^

The experimental results are average ± standard deviation. Duncan multiple range test showed that ^a–d^ and ^A–C^ of different letters showed significant difference in the mean value of each group (*P* < 0.05).

**Table 3:** Effects of lotus leaf-enriched flavonoid extract (LLEFE) on SOD, CAT activities, MDA, and GSH levels in HT-29 colon cancer cells.

Group		SOD (U/gport)	CAT (U/gport)	GSH (*μ*mol/mg)	MDA (nmol/gport)
Control		61.37 ± 4.47^d^	42.08 ± 3.96^d^	22.31 ± 3.31^d^	10.36 ± 1.12^a^
LLEFE (*μ*g/mL)	125	98.31 ± 5.11^c^	75.36 ± 4.09^c^	42.62 ± 3.88^c^	7.32 ± 0.55^b^
	250	148.38 ± 5.39^b^	125.67 ± 6.32^b^	70.36 ± 4.08^b^	4.69 ± 0.38^c^
	500	219.72 ± 7.70^a^	187.92 ± 5.83^a^	101.06 ± 5.36^a^	2.33 ± 0.28^d^

The experimental results are average ± standard deviation. Duncan multiple range test showed that ^a–d^ of different letters showed significant difference in the mean value of each group (*P* < 0.05).

## Data Availability

The datasets generated for this study are available upon request to the corresponding author.
